# Colorimetric Nanoparticle-Embedded Hydrogels for a Biosensing Platform

**DOI:** 10.3390/nano12071150

**Published:** 2022-03-30

**Authors:** Taeha Lee, Changheon Kim, Jiyeon Kim, Jung Bae Seong, Youngjeon Lee, Seokbeom Roh, Da Yeon Cheong, Wonseok Lee, Jinsung Park, Yoochan Hong, Gyudo Lee

**Affiliations:** 1 Department of Biotechnology and Bioinformatics, Korea University, Sejong 30019, Korea; xogk0038@korea.ac.kr (T.L.); kchh1018@korea.ac.kr (C.K.); marcia9812@korea.ac.kr (J.K.); 2017270446@korea.ac.kr (S.R.); 2017270450@korea.ac.kr (D.Y.C.); 2 Interdisciplinary Graduate Program for Artificial Intelligence Smart Convergence Technology, Korea University, Sejong 30019, Korea; 3 National Primate Research Center, Korea Research Institute of Bioscience and Biotechnology, Cheongju 28116, Korea; kks1613@kribb.re.kr (J.B.S.); neurosci@kribb.re.kr (Y.L.); 4 Department of Electrical Engineering, Korea National University of Transportation, Chungju 27469, Korea; wslee@ut.ac.kr; 5 Department of Biomechatronic Engineering, Sungkyunkwan University, Suwon 16419, Korea; 6 Department of Medical Device, Korea Institute of Machinery and Materials, Daegu 42994, Korea

**Keywords:** nanoparticle–hydrogel complex, colorimetric biosensor, agarose, polyaniline, pH sensor

## Abstract

Hydrogels containing colorimetric nanoparticles have been used for ion sensing, glucose detection, and microbial metabolite analyses. In particular, the rapid chemical reaction owing to both the hydrogel form of water retention and the sensitive color change of nanoparticles enables the rapid detection of target substances. Despite this advantage, the poor dispersibility of nanoparticles and the mechanical strength of nanoparticle–hydrogel complexes have limited their application. In this study, we demonstrate a milliliter agarose gel containing homogeneously synthesized polyaniline nanoparticles (PAni-NPs), referred to as PAni-NP–hydrogel complexes (PNHCs). To fabricate the optimal PNHC, we tested various pH solvents based on distilled water and phosphate-buffered saline and studied the colorimetric response of the PNHC with thickness. The colorimetric response of the prepared PNHC to the changes in the pH of the solution demonstrated excellent linearity, suggesting the possibility of using PNHC as a pH sensor. In addition, it was verified that the PNHC could detect minute pH changes caused by the cancer cell metabolites without cytotoxicity. Furthermore, the PNHC can be stably maintained outside water for approximately 12 h without deformation, indicating that it can be used as a disposable patch-type wearable biosensing platform.

## 1. Introduction

Nanoparticles (NPs) have been actively investigated over the past few decades because of their various physicochemical properties (such as conductivity, catalytic, and optical properties), depending on the material type and size [1,2,3,4,5,6]. NPs have been studied in materials science, biosensors, and nanomedicine as base materials, which are combined with other substances to develop novel materials [7,8,9,10,11]. To date, various NP-embedded complexes have been developed. Among them, NP-embedded hydrogel complexes have attracted considerable attention because of the advantages of hydrogels, such as biodegradability, biocompatibility and non-toxicity, which can be combined with the unique properties of NPs [12,13,14,15,16]. For example, Jung et al. demonstrated that the mechanical properties of a collagen hydrogel could be modulated by adding hydroxyapatite NPs [17]. Arno et al. demonstrated improved adhesion and resistance to breakage in calcium alginate hydrogels using poly(*L*-lactide)-based NPs [18]. Rose et al. reported an efficient method to assemble gels or tissues by combining silica NPs with polyacrylamide and poly(N,N-dimethylacrylamide) hydrogels [12]. Thakur et al. observed bacterial metabolism through color change using a complex film combining colorimetric polyaniline NPs (PAni-NPs) and agarose hydrogel [19]. Despite the numerous advantages of NP–hydrogel complexes, they have several limitations due to the non-uniform dispersibility of NPs in the hydrogel and the poor mechanical strength of the fabricated complex. Nevertheless, improving the functionality of the NP–hydrogel and maximizing its utility can be achieved by developing methods to overcome these limitations.

In this study, colorimetric PAni-NPs and agarose gel were combined to produce an advanced hydrogel complex exhibiting a rapid colorimetric response to environmental pH. Although PAni-NPs have a green color at low pH, they exhibit a blue color at higher pH [20]. Based on this unique colorimetric response, PAni-NPs have been widely used to monitor changes in the pH of the surrounding environment [21,22,23,24,25]. In addition, it serves as a suitable matrix for biomolecular immobilization and promotes electron transport during redox reactions [26,27]. Agarose gels are natural hydrogels with cross-linked hydrophilic substances that are insoluble in water. Hydrogels allow a rapid chemical reaction in the form of high water retention, biomimetic three-dimensional (3D) structure formation, high biocompatibility, and non-toxicity; therefore, many studies, such as multiplex assays, wearable patches, and biomolecule detection, are underway [28,29,30,31,32,33,34]. Consequently, the fabrication of the PAni-NP–hydrogel complex (PNHC) could simultaneously elicit the advantages of both PAni-NP and agarose gel. We thoroughly investigated the manufacture of an optimal PNHC with respect to volume, thickness, uniformity of surface morphology, cytotoxicity, colorimetric response to environmental pH, and stability outside water. We believe that our findings on PNHC can help in the development of new biosensing platforms based on colorimetric NP–hydrogel complexes.

## 2. Experimental Section

### 2.1. Materials

Aniline, pure ethyl alcohol, hydrochloric acid (HCl), ammonium persulfate (APS), pectin (extracted from apples), phosphate-buffered saline (PBS), Whatman 41 filter paper, agarose, distilled water (DW), dimethyl sulfoxide (DMSO), minimum essential medium (MEM), and Dulbecco’s modified Eagle’s medium (DMEM) were purchased from Sigma-Aldrich (Burlington, MA, USA). Penicillin–streptomycin from Cytiva (Marlborough, MA, USA), fetal bovine serum (FBS), RPMI medium 1640, 0.25% trypsin-EDTA (1X) from Gibco (Waltham, MA, USA), Type I bovine collagen solution from Advanced BioMatrix (Carlsbad, CA, USA), and streptomycin from Welgene (Gyeongsan, South Korea) were used. Polyethylene (PE) film and zinc diethyldithiocarbamate (ZDEC) were purchased from Hatano Research Institute (Hadano, Japan). 3-(4,5-Dimethylthiazol-2-yl)-2,5-diphenyl-2H-tetrazolium bromide (MTT) was purchased from Thermo Fisher (Waltham, MA, USA), and lactate dehydrogenase (LDH) was purchased from Promega (Madison, WI, USA).

### 2.2. Synthesis of PAni-NPs

PAni-NPs were synthesized using previously reported methods, with a few modifications [22]. A 25 mL APS solution at a 91.2 mg/mL concentration was added dropwise to a 60 mL solution containing 1.80 g pectin, 9 mL HCl (11 N), and 0.90 g aniline. The mixed solution was stirred for more than 4 h and then combined with a 1:1 mixture of pure ethanol and water to produce uniform NPs. Subsequently, PAni-NPs of uniform size were obtained using Whatman 41 filter paper.

### 2.3. Fabrication of PNHC

Agarose was diluted with DW to a concentration of 1%. The prepared agarose solution was sterilized by autoclaving at 120 °C for 15 min and then incubated at 23 °C for 5 min. The PAni-NP solution (20 mg/mL) was rapidly mixed with agarose solution at a 1:15 ratio. The prepared PAni-NP–hydrogel solution was dispensed into 6-well plates or Petri dishes (diameter: 35 mm) and was left at 23 °C for approximately 50 min until solidification.

### 2.4. Characterization

Images of PNHC were captured using a smartphone (Galaxy S20, Samsung, Suwon, South Korea) and a microscope (DS-Ri2, Nikon, Tokyo, Japan). The color of the PNHC was analyzed using ImageJ 1.53e software (NIH). The color information of the PNHCs was divided into three channels of colors (red, green, and blue) to quantify the color change from emeraldine base (EB) to emeraldine salt (ES), and the blue/green intensity ratio was calculated. The UV–visible spectra of the PAni-NPs and PNHCs were analyzed using a hybrid multimode reader (Synergy H1, BioTek, Winooski, VT, USA). The pH of the solutions was adjusted using a pH meter (Orion Star A211, Thermo Fisher Scientific, Waltham, MA, USA).

### 2.5. Cell Culture and Sample Extraction

HT-1080 fibrosarcoma human cell line was obtained from the Korean Cell Line Bank (Seoul, South Korea). The cells were maintained at 37 °C in RPMI 1640 supplemented with 20% FBS and 1% penicillin–streptomycin in a humidified 5% CO_2_ incubator. The L929 murine cell line was obtained from American Type Culture Collection (Manassas, VA, USA). The cells were maintained at 37 °C in MEM supplemented with 10% FBS and 1% penicillin–streptomycin in a humidified 5% CO_2_ incubator.

The samples were extracted following the ISO 10993 standards. High-density PE and polyurethane films containing 0.1% ZDEC were used as reference materials, where PE and ZDEC were used as a negative and positive control, respectively. PNHC extraction was performed in the culture medium, and PNHC, PE, or ZDEC were extracted at a ratio of 0.2 g/mL.

### 2.6. Analysis of Cell Viability and Cytotoxicity

Cell viability was analyzed using the MTT assay. The L929 cells were seeded at a density of 1 × 10^4^ cells/well in 96 well plates. Twenty-four hours after cell seeding, and the medium was exchanged with the treatment medium. After the incubation of the treatment medium for 45 h, 1 mg/mL of the MTT solution dissolved in PBS was added 10 µL of MTT solution to each well, and was incubated at 37 °C for 3 h in a CO_2_ incubator. The medium was carefully removed, and 100 µL of DMSO was added to each well to dissolve the formazan crystals. Absorbance was measured at 550 nm using a microplate spectrophotometer.

Cytotoxicity was also analyzed by measuring extracellular LDH release. After incubating for 48 h with the treatment medium in L929 cells, the supernatant was harvested, and the Cyto Tox 96 Non-radioactive cytotoxicity assay (LDH) was used to determine cell cytotoxicity. Absorbance was measured at 450 nm using a microplate spectrophotometer.

## 3. Results and Discussion

### 3.1. Fabrication of Biocompatible PNHC

**Figure 1:**
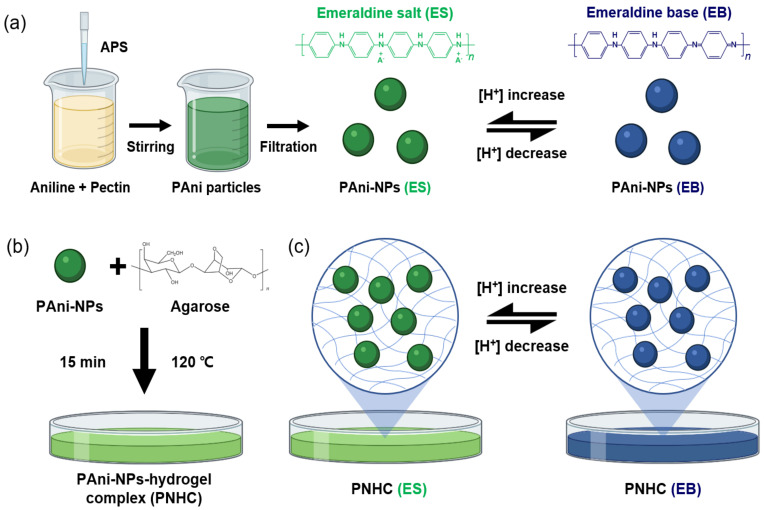
Schematic illustration of (**a**) the synthetic process and principle of color change of PAni-NPs with pH; (**b**) fabrication of PNHC by combining PAni-NPs and agarose; (**c**) principle of color change of the PNHC with pH. Abbreviations: ammonium persulfate (APS), polyaniline nanoparticles (PAni-NPs), PAni-NPs–hydrogel complex (PNHC), emeraldine salt (ES), and emeraldine base (EB).

The PAni-NPs were fabricated by the polymerization of aniline, and their colorimetric response to the surrounding pH was characterized (Figure 1a). The PAni-NPs capped by pectin had good stability in buffer solutions and exhibited effective color change with pH. The color of the PAni-NPs changed to green (ES state) at low pH (<pH 6) and to blue (EB state) at high pH (>pH 6). The PAni-NPs and agarose were mixed to produce PNHCs. During the PNHC incubation, a hydrogel network was gradually formed, and the PAni-NPs were embedded in the porous structure between the hydrogel networks (Figure 1b). We confirmed that the colorimetric response of PNHC is prompted by changes in the surrounding pH as much as the PAni-NP solution, implying that PAni-NPs are well dispersed in the hydrogel networks and undergo a stable redox reaction within PNHC (Figure 1c).

### 3.2. Optical Properties of PAni-NPs

**Figure 2:**
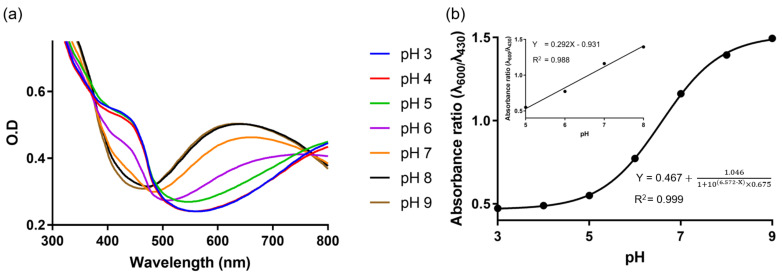
(**a**) Absorbance spectra of the PAni-NP solutions with varying pH. (**b**) Absorbance ratio (λ_600_/λ_430_) of the PAni-NP solutions from pH 3 to 9. Inset shows the absorbance ratio of PAni-NP solutions from pH 5 to 8, exhibiting good linearity.

We examined the quality of the synthesized PAni-NPs by spectrophotometric analysis of the absorbance peak corresponding to the π–π* transition of the benzenoid ring and polaron band. The ES state of the PAni-NPs changed to the EB state with the increasing pH; a decrease in absorbance was observed at 430 nm while an increase was observed at 600 nm (Figure 2a). Next, we plotted the absorbance ratio (λ_600_/λ_430_) of PAni-NPs vs. the pH of the solution ranging from 3 to 9 (Figure 2b). An excellent linearity is observed, particularly at pH 5–8 (as shown in the inset of Figure 2b), which is consistent with the results of our previous study [22]. It should be noted that the linear range of PAni-NPs for the colorimetric response is sufficient for various biomedical applications.

### 3.3. Physical Properties of PNHC

**Figure 3:**
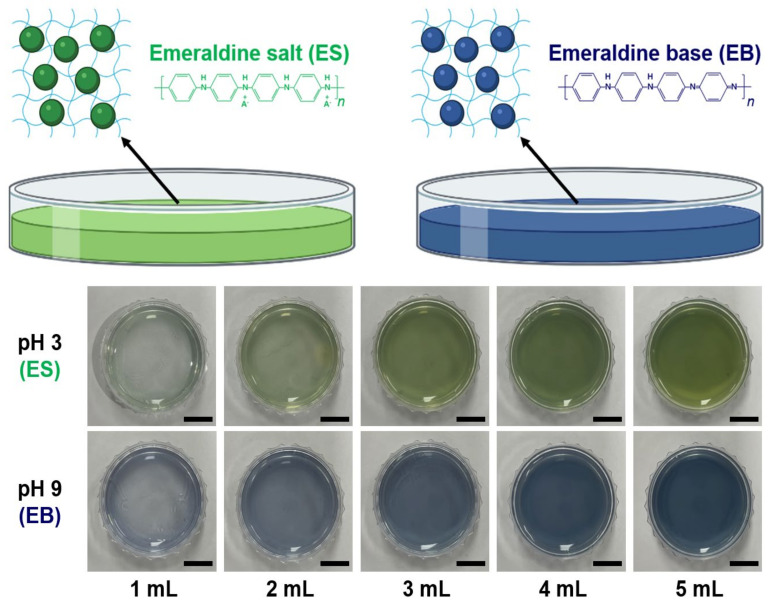
Molecular structure inside the PNHC with pH, and the effect of volume on the colorimetric properties of PNHC. Schematic illustration of the molecular structure inside the PNHC fabricated at different pH (3 and 9). Images of PNHCs with different volumes (1, 2, 3, 4 and 5 mL) at pH 3 and 9. Scale bar represents 1 cm.

The physical properties (thickness and shape) of the PNHCs are investigated with different amounts of PAni-NP–hydrogel solutions (1, 2, 3, 4, and 5 mL), as shown in Figure 3. The shape of the PNHCs was determined by crosslinking the hydrogel solution in a 35 mm diameter Petri dish. All fabricated PNHCs exhibited an excellent color change from the ES state (green) to the EB state (blue) and vice versa. However, the colorimetric reaction of the PNHCs prepared at low volumes (1 and 2 mL) was relatively difficult to confirm because of their non-uniform surface and thin thickness. In contrast, the surface was relatively uniform in the case of high volumes (4 and 5 mL); however, the colorimetric reaction was comparatively slow owing to the thickness of the hydrogel. Therefore, the hydrogel volume was optimized to 3 mL under the experimental conditions to prepare PNHCs with a uniform surface and appropriate thickness.

The PNHC with the optimal thickness can completely change color from EB to ES within 8 min. To examine the transition ability of PNHC in the redox reaction, we implemented a colorimetric response test in which low-pH and high-pH solutions were sequentially changed three times (Figure S1 and Supplementary Movie S1). Subsequently, PNHC was kept in room conditions (23 °C, 29% in relative humidity) for 12 h to investigate its stability; however, the point of change was barely identified. This result implies that the abundant water molecules inside the hydrogel ensured long-term stability within the PNHC (Figure S2).

**Figure 4:**
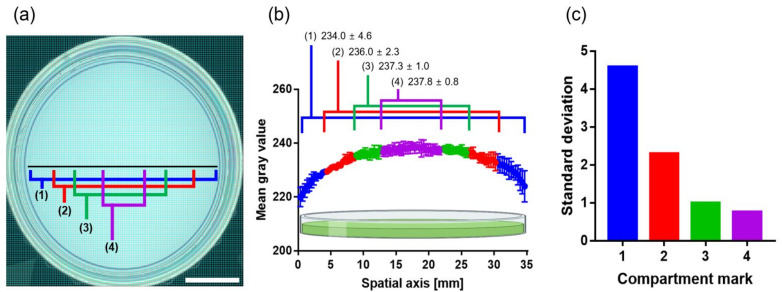
(**a**) Image of PNHC with compartment marks to investigate the thickness variation of the PNHC across the dish. Scale bar represents 1 cm. (**b**) Mean gray values with standard deviation corresponding to compartment mark 1–4 are co-plotted along the spatial axis (35 mm) (*n* = 3). (**c**) Standard deviations of each compartment mark.

Because the hydrogel has strong water properties, the PNHC cast in a Petri dish has a meniscus. This meniscus suggests several variations in the thickness of the PNHC across the plate. This thickness variation can lead to errors when analyzing the colorimetric response of the PNHC. Therefore, to investigate the relationship between the thickness and the color intensity of the PNHC, we divided the location of the PNHC into four different compartments in the radial direction from the center (Figure 4a). The mean gray value of each compartment was extracted using the ImageJ software. The average gray value represents the contrast of the PNHC, while a smaller value indicates a thicker PNHC. In particular, the contrast was inversely proportional to the hydrogel thickness. We plotted the average gray values for each compartment (from 1 to 4) along with the diameter of the PNHC (Figure 4b). The measurement results showed that the contrast of the PNHC increased and became uniform as it approached the center. Conversely, the thickness of the PNHC became uniform as it approached the center, similar to a volcanic crater.

To quantify the uniformity of the surface morphology of the PNHC, we calculated the standard deviations of the average gray values from each compartment (1–4): 4.6% for #1, 2.3% for #2, 1.0% for #3, and 0.8% for #4 (Figure 4c). Collectively, the formation of meniscus is inevitable when a milliliter of PNHC is manufactured in a Petri dish. Nevertheless, we validated that the analysis error could be significantly reduced by analyzing the colorimetric response near the center (compartments 3 or 4).

### 3.4. Effect of Solvent Type on Intrinsic Color of PNHC

**Figure 5:**
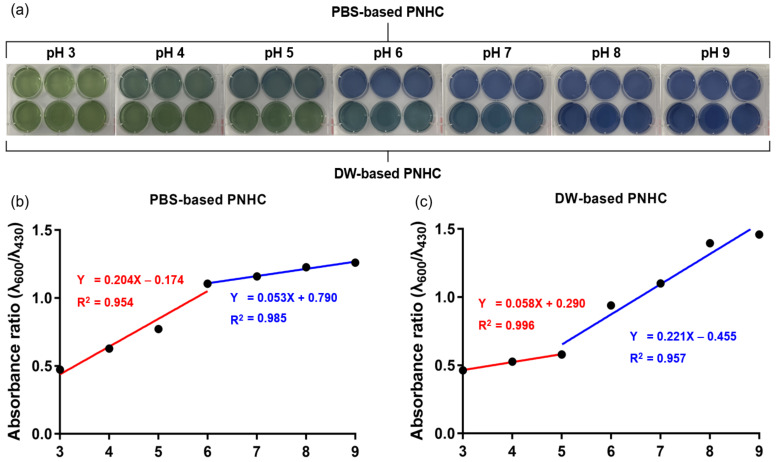
Effect of the solvent type and pH on the intrinsic color of PNHC. (**a**) Images for 14 different types of PNHCs that were fabricated based on PBS (pH 3–9) and DW (pH 3–9). (**b**) Absorbance ratio (λ_600_/λ_430_) of the PBS-based PNHC and (**c**) DW-based PNHC due to the pH of the solvent used in the PNHC fabrication. For all the data point, the error bars are shorter than the symbol height.

To investigate the influence of the solvent used for the fabrication of PNHC on the quality of the PNHC, we characterized the intrinsic color of the PNHC composed of PBS and DW at different pH levels (Figure 5a). In particular, we fabricated 14 different types of PNHC by solvent type and pH (3–9) and analyzed their spectrophotometric features. Regardless of the solvent used, the overall trend was visibly similar, that is, the ES and EB states at low and high pH, respectively. However, segmental linear regression, an interesting feature, was observed in the result of the absorbance ratio analysis. As shown in Figure 5b, PBS-based PNHC exhibits an evident color change in the low pH range (pH 3–6), which is not observed in the high pH range (pH 6–9). The slope of the absorbance ratio according to the pH was 0.204 and 0.053 pH^−1^ for pH 3–6 and 6–9, respectively. In contrast, the DW-based PNHC demonstrated a smaller slope (0.058 pH^−1^) and larger slope (0.221 pH^−1^) for the color change in the low pH range (pH 3–5) and high pH range (pH 5–9), respectively (Figure 5c). Consequently, the colorimetric properties of the PNHC drastically changed between pH 5 and 6. This segmental linear regression of data can be attributable to the dynamic colorimetric characteristic of the PAni-NPs within the PNHC, which is determined by combining the swelling responses of the agarose gel to pH and the degree of buffering effect of the main solvent. Further in-depth studies are required to understand this phenomenon fully [35,36,37]. Nevertheless, our findings suggest that the static and dynamic colorimetric properties of PNHC can be modulated to suit the purpose by adjusting the type and pH of the primary solvent.

### 3.5. Colorimetric Responses of PNHC to pH

**Figure 6:**
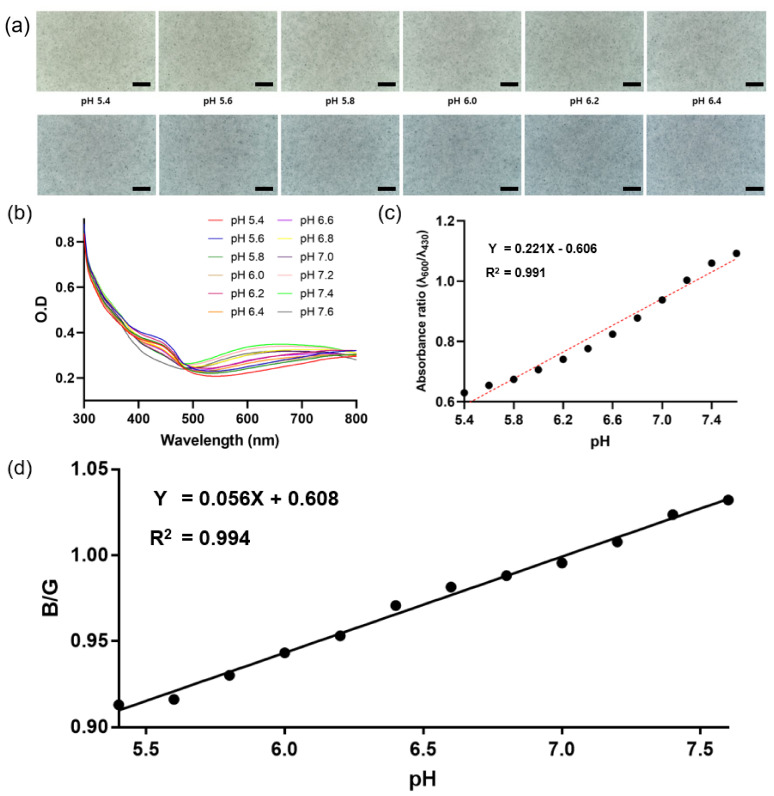
Colorimetric response of PNHC with the surrounding pH. (**a**) Microscopic images of the PNHC surface exposed to various pH conditions (from 5.4 to 7.6). Scale bar represents 200 μm. (**b**) Absorbance spectra of the PNHCs at specific wavelengths changed by pH. (**c**) Absorbance ratio (λ_600_/λ_430_) of the PNHC with pH conditions. (**d**) B/G intensity ratio of PNHC vs. pH. The relative signal intensity (B/G) is a ratio of the blue value divided by the green value, which is quantified using ImageJ software.

To estimate the performance of the PNHC as a colorimetric pH biosensor, we investigated the colorimetric response of the PNHC at specific pH levels. In particular, we obtained the microscopic images of the PNHCs at 0.2 pH intervals in physiologically relevant pH ranges (5.4–7.6) (Figure 6a). A clear green color was observed at lower pH, and blue color was confirmed at higher pH. These variations of color in the PNHC were quantified by spectrophotometric analysis. In the absorbance spectrum of PNHC vs. pH (Figure 6b), the intensity at 430 nm (λ_430_) decreased and that at 600 nm (λ_600_) increased as the pH increased. By calculating the absorbance ratio (λ_600_/λ_430_) of PNHC, we could better understand the colorimetric response of the PNHC to pH (Figure 6c). In the pH range of 5.4 to 7.6, the absorbance ratio was proportional to the pH (R^2^ = 0.991). The sensitivity of the sensor developed by Thakur et al. is about 0.13 [19], while what we developed is about 0.22, which is about twice better. However, Thakur et al. did not use the absorbance ratio but use absorbance intensity at a specific wavelength (λ_420_), so it cannot be accurately compared with our results. Since the colorimetric sensitivity can vary depending on the concentration and quality of PAni-NPs embedded in the hydrogel, we believe that it could be further improved through the optimization of the PNHC for practical sensing. Subsequently, we extracted the blue (B) and green (G) values from the PNHC images and calculated the B/G ratio. This process is important for PNHC-based colorimetric pH sensing in the absence of spectrophotometry. Figure 6d shows a plot of the B/G values of PNHC vs. the surrounding pH, exhibiting better linearity (R^2^ = 0.994) compared to the absorbance ratio-based analysis. This result suggests that the pH of the solution treated on the PNHC can be inversely estimated by calculating the B/G factor from the PNHC image.

### 3.6. Application of PNHC as Biosensing Platforms

**Figure 7:**
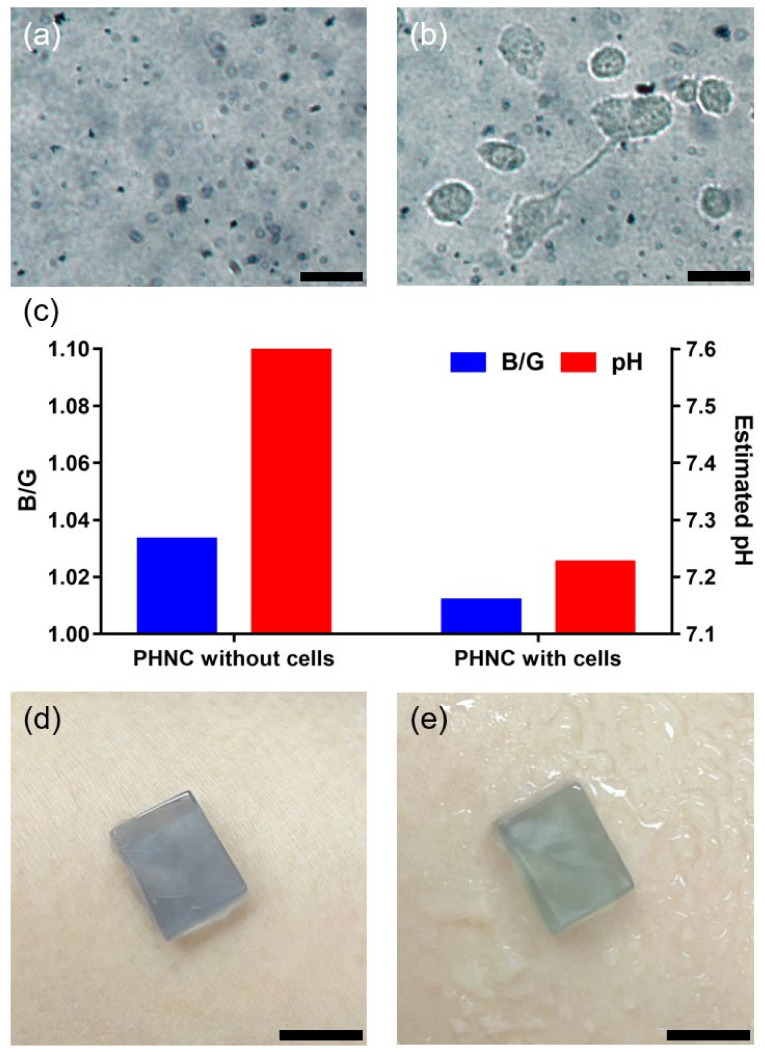
(**a**) PNHC without cells and (**b**) PNHC with cell for 6 h after cell attachment. Scale bar represents 40 µm. (**c**) B/G of PNHC without and with cells and estimated pH from the B/G values using the model equation in Figure 6d. The signal intensity (B/G) is a ratio of the blue value divided by the green value; the color intensities were quantified using ImageJ software. Photographs of (**d**) the PNHC on the native skin of a human arm and (**e**) the PNHC after spraying artificial sweat solution. Scale bar represents 5 mm.

Based on the superior colorimetric response of the PNHC to pH, we explored the application of PNHC. First, we tested whether PNHC could detect minute pH changes in cell metabolites. The adherent cells were incubated on the PNHC surface for direct colorimetric testing. Prior to incubation, we performed a cytotoxicity test for PNHC and validated that it was not cytotoxic (Figure S3). Accordingly, HT-1080 cells were incubated on PNHC for 6 h, and we analyzed the images of PNHC before and after treatment (Figure 7a,b). We confirmed that several cells were attached well and were largely elongated on the PNHC surface, indicating that the cells were under active metabolism (for example, glycolysis) [38]. The B/G values were extracted from around the attached cells in the image and compared with those of the bare PNHC. The B/G from the PNHC with cells was lower than that from the PNHC without cells as a negative control (Figure 7c). This result implies that pH decreased upon the PNHC incubated cells with PNHC due to cellular metabolites (such as lactate and pyruvate). In contrast, the pH rarely changed in PNHC incubated in the cell culture medium without cells. Using the model depicting the relationship between the B/G factor and the surrounding pH (Figure 6d), we estimated the pH values for PNHC with and without cells. PNHCs without cells were estimated to have a pH of 7.6, while PNHCs with cells were assessed to have a pH of 7.2. The decrease in pH by only 0.4 is relatively reasonable because HT-1080 is well known as a malignant cancer cell; however, it depends on the oxidative phosphorylation and not glycolysis during the energy production process [39,40]. These results suggest the practicality of PNHC for monitoring the pH of live cells.

We used PNHC as a wearable biosensor, such as a pH patch, to examine its further application. As shown in Figure 7d, the PNHC wearable patch was manufactured in 0.8 cm × 1.2 cm × 0.3 cm in width, length, and height. Because of the excellent physical properties of PNHC, that is, its high mechanical strength and adhesion ability to the skin, it can be used as an attachable patch-type wearable biosensor [41,42,43]. Furthermore, the PNHCs maintained their shape and color stability while being attached to the skin (Figure 7d). Moreover, the color of the PNHC changed to green when artificial sweat (pH 6) was sprayed near the PNHC (Figure 7e). These results imply that the PNHC can be used as a patch-type wearable biosensor for monitoring the pH of the sweat.

## 4. Conclusions

In this study, we developed a PNHC that detects the surrounding pH effectively. We optimized the quality and performance of the PNHC by various experiments with respect to solvent type and hydrogel volume. The PNHC was fabricated with a uniform surface and good dispersibility of the PAni-NPs. The PNHC exhibited an outstanding performance for pH sensing based on the advantage of high water-retention with a 3D networking structure and the rapid doping/dedoping transition of the PAni-NPs under a redox reaction. Moreover, with the high biocompatibility and non-toxicity of PNHC, we were able to detect pH changes in living cells cultured on the PNHC surface. Furthermore, because of the moisturized surface of PNHC, it can be well attached to the skin and shows rapid color transition with pH change. Based on our results, PNHC, as an advanced version of the colorimetric nanoparticle-embedded hydrogel complex, shows strong potential for use in various biosensing platforms.

## Supplementary Materials

The following supporting information can be downloaded at: https://www.mdpi.com/article/10.3390/nano12071150/s1. Figure S1. Reversible test of PNHC through absorbance ratio (λ_600_/λ_430_). The absorbance was measured in a microplate spectrophotometer. The pH of the solution was varied every 40 min; Figure S2. Retention of PNHC morphology outside water. PNHC was observed after 12 h; Figure S3. (a) MTT and (b) LDH assays in L929 cells. The absorbance of the MTT formazan and LDH activity was determined at 550 nm and 490 nm, respectively, in a microplate spectrophotometer. Supplementary Movie S1.
