# Supplementary material for: Colorimetric Nanoparticle-Embedded Hydrogels for a Biosensing Platform
Source: Nanomaterials (Basel). 2022 Mar 30;12(7):1150. doi: 10.3390/nano12071150 (PMC9000776; doi:10.3390/nano12071150)
Supplement: Supplementary file 1 [file nanomaterials-12-01150-s001.zip › nanomaterials-1654822SI.pdf]

nanomaterials

Supplementary Information

Colorimetric Nanoparticle-Embedded Hydrogels for a Biosensing Platform

Taeha Lee [1,2,†], Changheon Kim [1,†], Jiyeon Kim [1,†], Jung Bae Seong [3], Youngjeon Lee [3], Seokbeom Roh [1,2], Da Yeon Cheong [1,2], Wonseok Lee [4], Jinsung Park [5,*], Yoochan Hong [6,*] and Gyudo Lee [1,2,*]

[1] Department of Biotechnology and Bioinformatics, Korea University, Sejong 30019, Korea; xogk0038@korea.ac.kr (T.L.); kchh1018@korea.ac.kr (C.K.); marcia9812@korea.ac.kr (J.K.); 2017270446@korea.ac.kr (S.R.); 2017270450@korea.ac.kr (D.Y.C.)
[2] Interdisciplinary Graduate Program for Artificial Intelligence Smart Convergence Technology, Korea University, Sejong 30019, Korea
[3] National Primate Research Center, Korea Research Institute of Bioscience and Biotechnology, Cheongju 28116, Korea; kks1613@kribb.re.kr (J.B.S.); neurosci@kribb.re.kr (Y.L.)
[4] Department of Electrical Engineering, Korea National University of Transportation, Chungju 27469, Korea; wslee@ut.ac.kr
[5] Department of Biomechatronic Engineering, Sungkyunkwan University, Suwon 16419, Korea
[6] Department of Medical Device, Korea Institute of Machinery and Materials, Daegu 42994, Korea
* Correspondence: nanojspark@skku.edu (J.P.); ychong1983@kimm.re.kr (Y.H.); lkd0807@korea.ac.kr (G.L.)
† These authors contributed equally to this work.

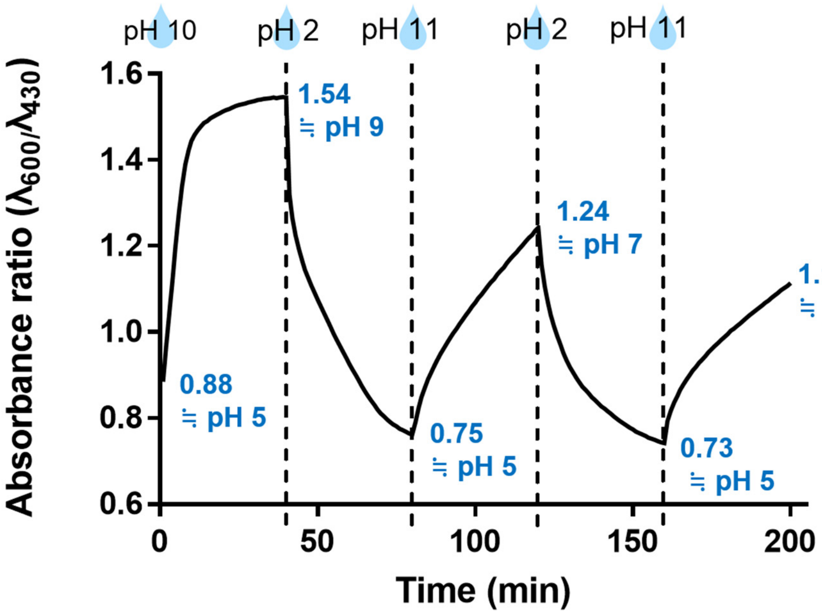

Figure S1. Reversible test of PNHC through absorbance ratio ($\lambda_{600}/\lambda_{430}$). The absorbance was measured in a microplate spectrophotometer. The pH of the solution was varied every 40 min.

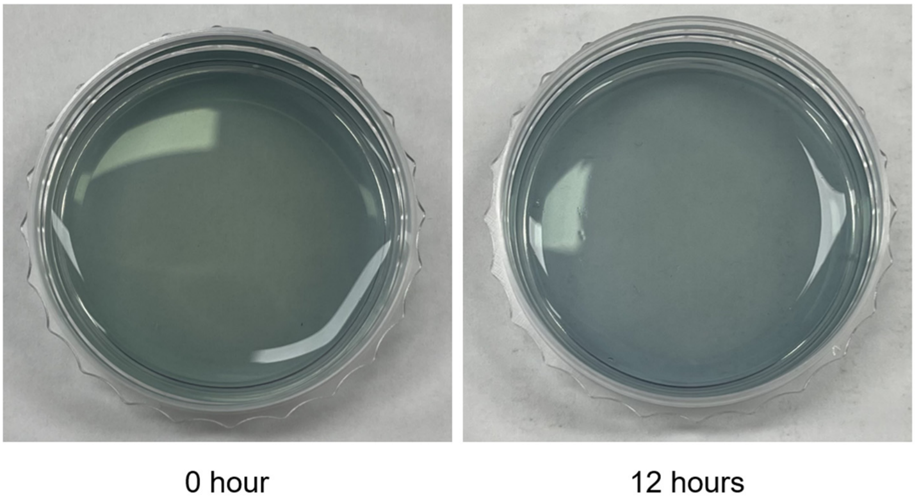

Figure S2. Retention of PNHC morphology outside water. PNHC was observed after 12 h.

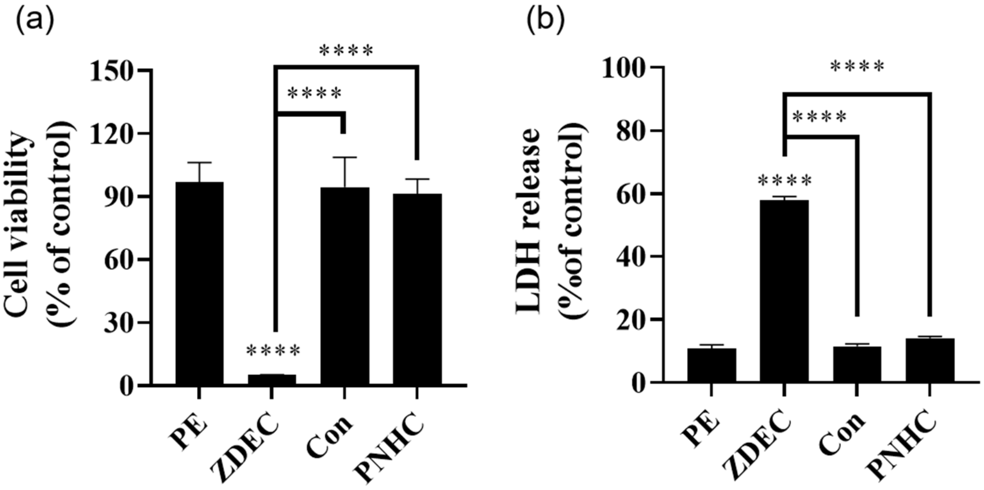

Figure S3. (**a**) MTT and (**b**) LDH assays in L929 cells. The absorbance of the MTT formazan and LDH activity was determined at 550 nm and 490 nm, respectively, in a microplate spectrophotometer.
