# Supplementary figures and images for: Colorimetric Nanoparticle-Embedded Hydrogels for a Biosensing Platform
Source: Nanomaterials (Basel). 2022 Mar 30;12(7):1150. doi: 10.3390/nano12071150 (PMC9000776; doi:10.3390/nano12071150)

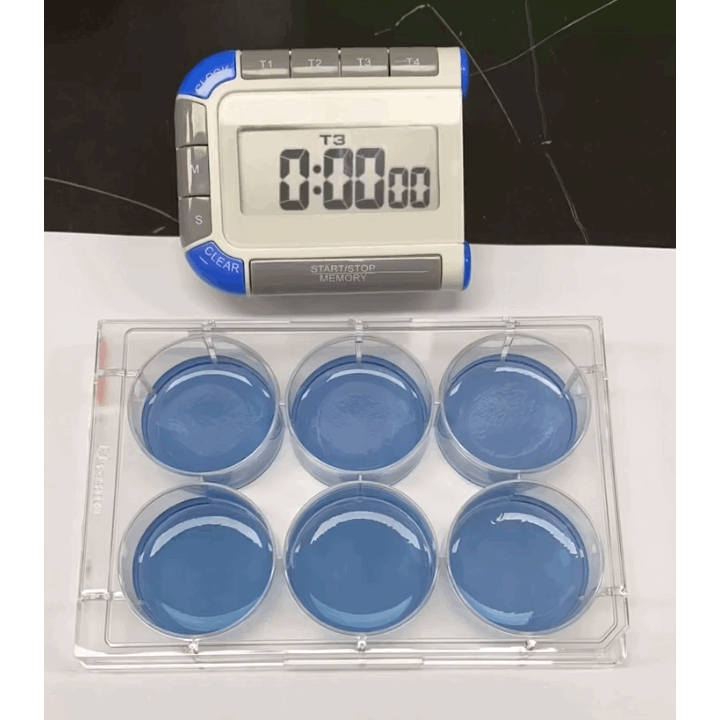

Supplement: Supplementary file 1 [file nanomaterials-12-01150-s001.zip › Supplementary Movie S1.gif]
